# Integrated multi-omics analysis and machine learning refine molecular subtypes and clinical outcome for hepatocellular carcinoma

**DOI:** 10.1186/s41065-025-00431-6

**Published:** 2025-04-12

**Authors:** Chunhong Li, Jiahua Hu, Mengqin Li, Yiming Mao, Yuhua Mao

**Affiliations:** 1 Central Laboratory, Guangxi Health Commission Key Laboratory of Glucose and Lipid Metabolism Disorders, The Second Affiliated Hospital of Guilin Medical University, Guilin, 541199 Guangxi China; 2 Guangxi Health Commission Key Laboratory of Glucose and Lipid Metabolism Disorders, The Second Affiliated Hospital of Guilin Medical University, Guilin, 541199 Guangxi China; 3 College of pharmacy, Guilin Medical University, Guilin, 541199 Guangxi China; 4 Department of thoracic surgery, Suzhou Kowloon Hospital, Shanghai Jiao Tong University School of Medicine, Suzhou, 215028 China; 5 Department of Obstetrics, The Second Affiliated Hospital of Guilin Medical University, Guilin, 541199 Guangxi China

**Keywords:** Hepatocellular carcinoma, Molecular typing, Machine learning, Tumor microenvironment, Immunotherapy

## Abstract

The high morbidity and mortality of hepatocellular carcinoma (HCC) impose a substantial economic burden on patients’ families and society, and the majority of HCC patients are detected at advanced stages and experience poor therapeutic outcomes, whereas early-stage patients exhibit the most favorable prognosis following radical treatment. In this study, we utilized a computational framework to integrate multi-omics data from HCC patients using the latest 10 different clustering algorithms, which were then employed a diverse set of 101 combinations derived from 10 different machine learning algorithms to develop a consensus machine learning-based signature (CMLBS). Using multi-omics consensus clustering, we distinguished two cancer subtypes (CSs) of HCC, and found that CS2 patients exhibited superior overall survival (OS) outcomes. In TCGA-LIHC, ICGC-LIRI, and multiple immunotherapy cohorts, low-CMLBS patients demonstrated favorable clinical outcomes and enhanced responsiveness to immunotherapy. Encouragingly, we observed that the high-CMLBS patients may exhibit increased sensitivity to Alpelisib, AZD7762, BMS-536,924, Carmustine, and GDC0810, whereas they may demonstrate reduced sensitivity to Axitinib, AZD6482, AZD8055, Entospletinib, GSK269962A, GSK1904529A, and GSK2606414, suggesting that CMLBS may contribute to the selection of chemotherapeutic agents for HCC patients. Therefore, in-depth examination of data from multi-omics data can provide valuable insights and contribute to the refinement of the molecular classification of HCC. In addition, the CMLBS model demonstrates potential as a screening tool for identifying HCC patients who may derive benefit from immunotherapy, and it possesses practical utility in the clinical management of HCC.

**Supplementary Information:**

The online version contains supplementary material available at 10.1186/s41065-025-00431-6.

## Introduction

Primary liver cancer is one of the six most prevalent solid tumors worldwide and the third dominating cause of tumor-related mortality, exhibiting high morbidity and mortality rates [1]. It primarily comprises three pathological forms: hepatocellular carcinoma (HCC), intrahepatic cholangiocarcinoma (ICC), and combined hepatocellular carcinoma-cholangiocarcinoma, of which approximately 80% are hepatocellular carcinomas [2, 3]. Roughly 70% of HCC patients are identified at intermediate to advanced stages, precluding the optimal opportunity for surgical resection [4]. In recent years, with the significant advancement of immunotherapy in the treatment of malignant tumors, immunotherapy represented by immune checkpoint inhibitors (ICIs), primarily including PD-1/PD-L1 inhibitors and CTLA-4 inhibitors, has demonstrated efficacy in the intervention of advanced HCC patients [5–7], but not all patients achieve favorable therapeutic outcomes. In addition, despite these substantial advancements, the molecular mechanisms through which immunotherapy modulates the immune microenvironment and regulates immune responses and evasion in HCC patients remain incompletely elucidated [5]. Therefore, a critical challenge lies in identifying HCC patients who may derive benefit from checkpoint inhibitor therapy.

In recent years, with the rapid advancement of multi-omics technologies, the molecular typing system for HCC has been established, such as liver-specific gene signature [8], neutrophil-derived signature [9], immune-related cell death signature [10], ferroptosis-related signature [11], and N6-methylandenosine-related signature [12]. Building upon this foundation, the integration of multi-omics features enables further refinement of the classification system, facilitating the identification of subtypes characterized by distinct molecular features and clinical prognoses. Investigations into the genome, transcriptome, proteome, and phosphorylation and lactylome of HCC have enhanced our understanding of HCC biology, contributing to the development of molecular typing for early-stage HCC and the exploration of mechanisms underlying its progression and potential drug targets for treatment [13–15]. Through the integration of proteomic, metabolomic, and lipidomic data, the reference spectrum of HCC subtypes has been successfully established, which may facilitate more precise and personalized treatment strategies for HCC patients [16]. In addition, certain studies have elucidated the molecular typing, prognostic features, and therapeutic targets of HCC patients from the perspective of proteomics, phosphoproteomics, and ubiquitomics, providing a significant theoretical foundation for the prognosis and assessment of HCC [17]. Although molecular typing presents novel opportunities for precision treatment of HCC, several challenges persist, including the accurate typing of HCC, the appropriateness of perioperative immunological application, the differential benefits of immunotherapy for HCC patients with varying etiologies, and the paucity of data from large-scale clinical trials. Consequently, the widespread clinical application of these approaches remains premature, necessitating the development of more refined molecular models and novel drugs targeting distinct molecular subtypes. In this study, we employed 10 different clustering algorithms to establish an integrated molecular subtype of HCC by incorporating data from mRNA, long non-coding RNA (lncRNA), microRNA (miRNA), epigenomic DNA methylation expression profiles, and genomic mutations. We then detected 145 prognosis-related genes (PRGs) based on their differential expression analysis between the two subtypes and utilized 10 different machine learning algorithms to develop a CMLBS model. Finally, we evaluated the efficacy of the CMLBS in terms of clinical pathological features, prognosis, and its potential use in immunotherapy and targeted chemotherapy. Based on the findings, this study provides a crucial reference for enhancing HCC molecular subtypes and improving precision and personalized treatment strategies for HCC patients.

## Results

### Multi-omics integrative molecular subtype of hepatocellular carcinoma

**Fig. 1:**
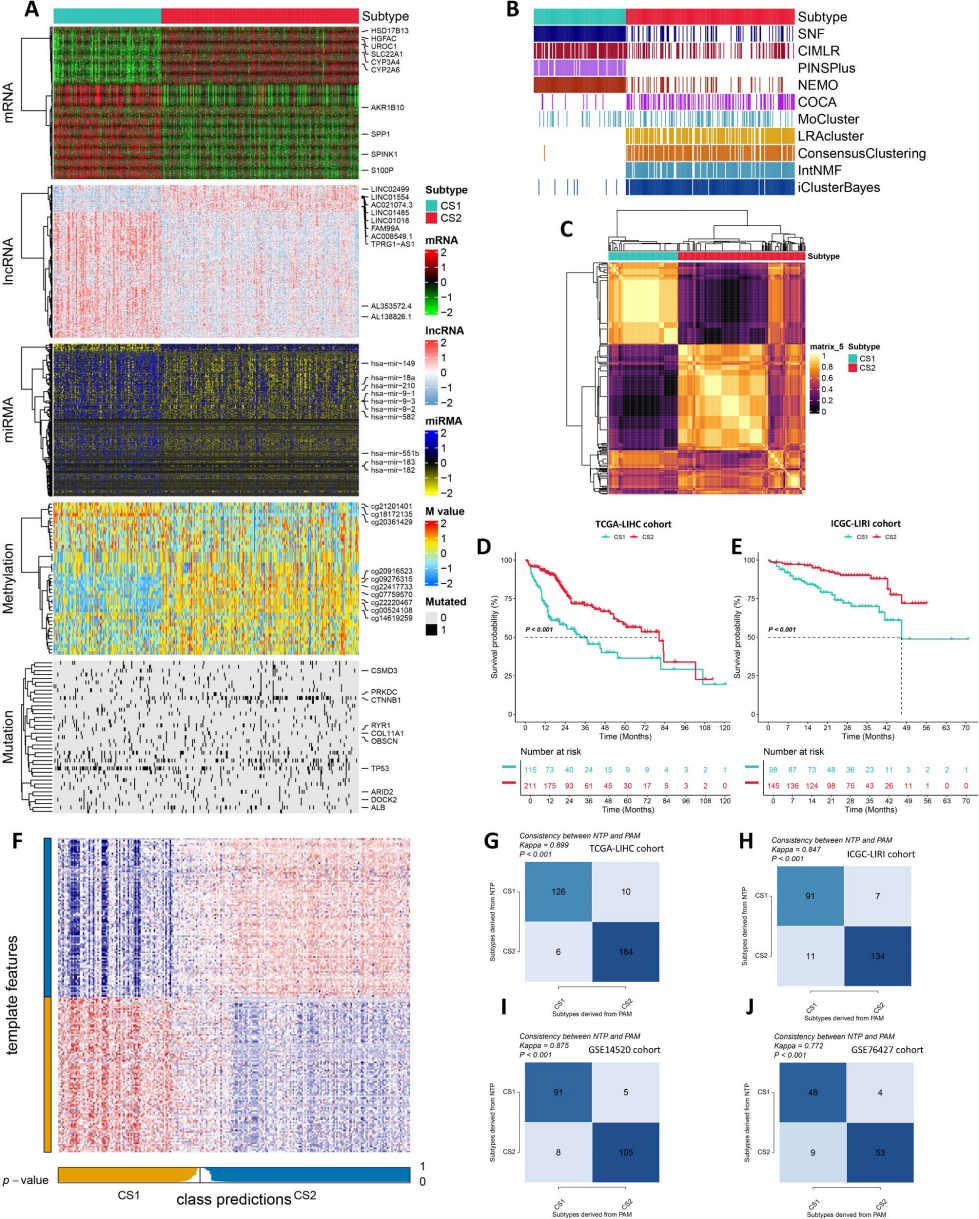
Multi-omics integrative molecular subtype of hepatocellular carcinoma. (**A**). Comprehensive heatmap of consensus ensemble subtypes, including mRNA, lncRNA, miRNA, DNA CpG methylation site, and mutant gene. (**B**). Clustering of HCC patients through 10 cutting-edge multi-omics clustering methods. (**C**). Consensus clustering matrix for two novel prognostic subtypes based on the 10 algorithms. (**D**). Different survival outcomes among the two subtypes in the TCGA-LIHC cohort. (**E**). Different survival outcomes among the two subtypes in the ICGC-LIRI cohort. (**F**). Validation of HCC CSs in the nearest template of the ICGC-LIRI cohort. (**G**). The consistency of NTP with PAM in the TCGA-LIHC cohort. (**H**). The consistency of NTP with PAM in the ICGC-LIRI cohort. (**I**). The consistency of NTP with PAM in the GSE14520 cohort. (**J**). The consistency of NTP with PAM in the GSE76427 cohort

Through the evaluation of 10 distinct clustering statistics, gap statistical analysis, cluster prediction index, silhouette score, and established molecular classifications, we concluded that two was the optimal number of clusters (Figures S1 and S2). Subsequently, two cancer subtypes (CSs) were identified through integrative clustering analysis, which revealed distinct molecular patterns across various profiles, including transcriptome (mRNA, lncRNA, and miRNA), epigenomic DNA methylation, and somatic mutations (Fig. 1A and C). The survival outcomes exhibited a statistically significant association with our classification system (*P* < 0.001; Fig. 1D). Interestingly, among the two clusters, cancer subtype 2 (CS2) demonstrated the best overall survival (OS) rates. Utilizing the NTP classifies, each patient in the outside cohort is classified into one of the identified CSs. This corresponds with the observation that CS2 in the ICGC-LIRI cohort demonstrated the most favorable prognosis among all subtypes (*P* < 0.001, Fig. 1E and F). The consistency between NTP and PAM was also assessed, yielding comparable outcomes in the TCGA-LIHC, ICGC-LIRI, GSE14520, and GSE76427 cohorts (*P* < 0.001, Fig. 1G and J).

### Molecular characterization of hepatocellular carcinoma subtypes

**Fig. 2:**
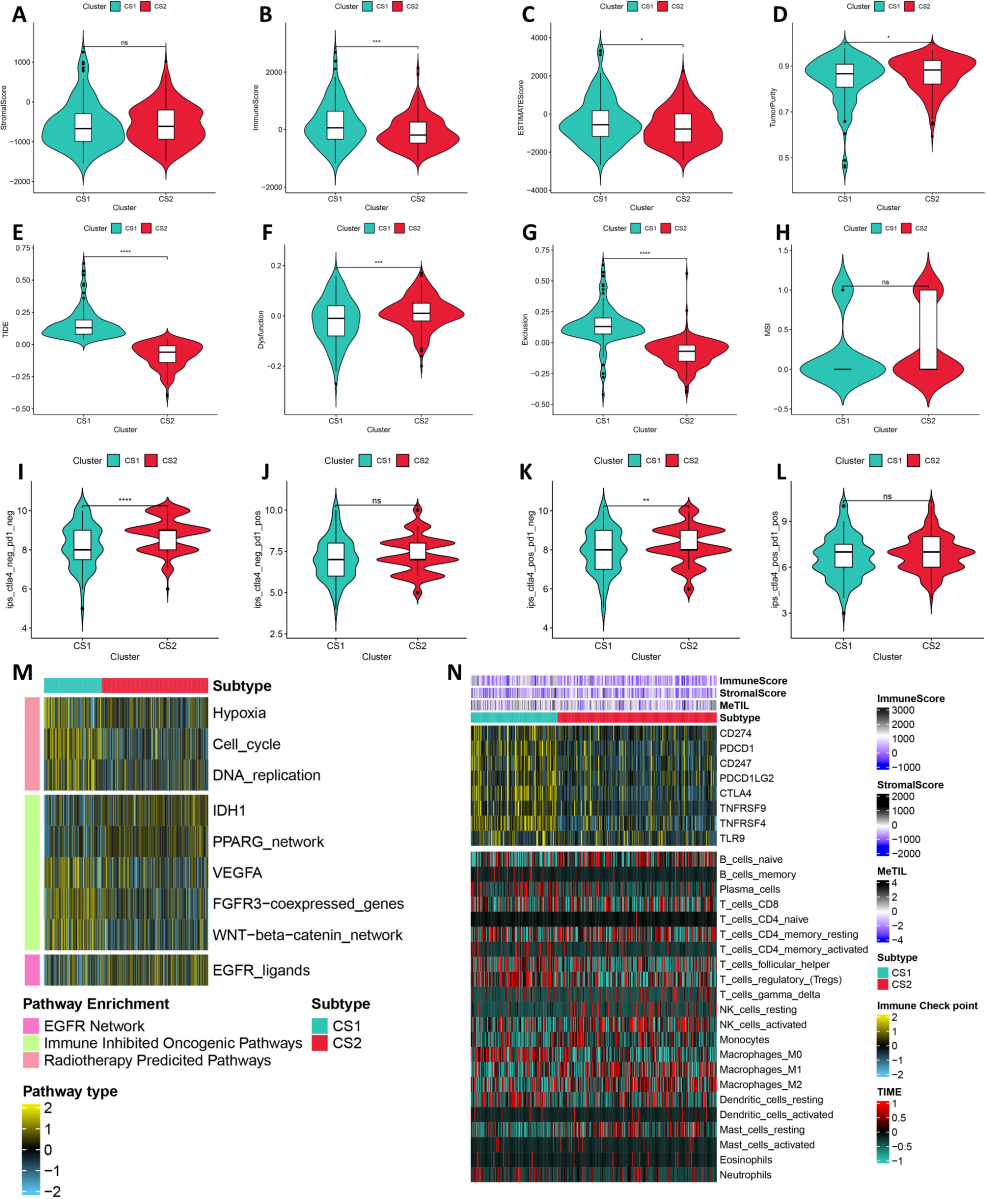
Molecular characterization of hepatocellular carcinoma subtypes. (**A**-**D**). Stroma score, Immune score, ESITIMATE score, and Tumor purity in the two subtypes. (**E**-**H**). TIDE score, T-cell dysfunction score, T-cell rejection score and microsatellite instability (MSI) score in the two subtypes. (**I**-**L**). Comparisons of the IPS in the two subtypes. (**M**). The enrichment of two subtypes for different treatment-related signatures. (**N**). Immune profiles in the TCGA-LIHC cohort. The top annotation of the heatmap shows the immune enrichment score, stromal enrichment score, and DNA methylation of tumor-infiltrating lymphocytes. The top panel shows the expression of canonical immune checkpoint genes, and the bottom panel shows the enrichment levels of 24 TME-related immune cells

To further elucidate the heterogeneity of these identified subtypes, we executed a all-inclusive analysis. The results acquired from the ESITIMATE algorithm indicated that CS1 patients exhibited elevated estimated scores and immune scores, while demonstrating reduced stromal scores and tumor purity (Fig. 2A and D). The results obtained from the TIDE algorithm indicated that CS1 patients exhibited elevated TIDE scores and T cell exclusion scores, while demonstrating reduced T cell dysfunction scores and microsatellite instability (MSI) scores, which suggests an enhanced capacity for immune evasion in CS1 patients and potentially diminished efficacy of immune checkpoint inhibitor (ICI) treatments (Fig. 2E and H). The results of the immunophenoscore indicated that immune checkpoint inhibitor therapy demonstrated limited therapeutic efficacy in CS1 patients (Fig. 2I and L). Furthermore, significant variations in response to specific therapies were observed among identified subtypes. CS1 patients exhibited a higher likelihood of benefiting from therapies such as radiotherapy-predicted pathways, whereas pathways such as the EGFR Network were significantly enriched in CS2 patients (Fig. 2M). Considering the crucial importance of tumor immunity in cancer development and progression, we assessed the levels of infiltrating microenvironmental cells. Our results revealed a substantial increase in immune cell infiltration in CS1 patients, while CS2 patients exhibited comparatively lower levels (Fig. 2N and Figure S3). Promising IC50 results were noted, showing enhanced efficacy for conventional chemotherapy drugs in CS2 patients relative to CS1 patients (Figure S4).

### Integrative construction of a consensus machine learning-based prognostic signature

**Fig. 3:**
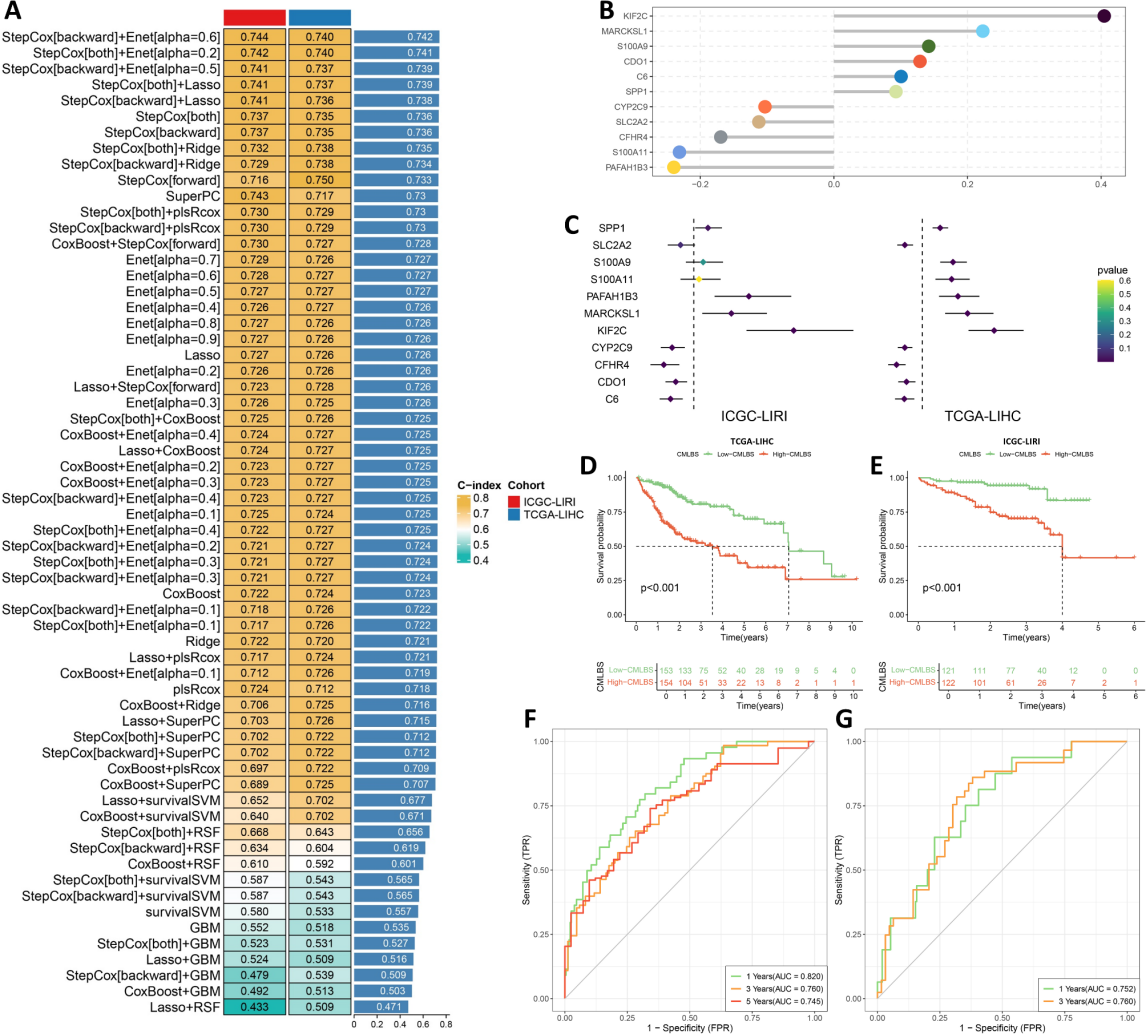
Integrative construction of a consensus machine learning-based prognostic signature. (**A**). A total of 101 different prediction models by our machine learning computational framework and further calculated the C-index of each model in the validation cohort. (**B**). The hub gene selected through the StepCox [backward] and Enet [alpha = 0.6] algorithm. (**C**). The univariate Cox regression analysis results of hub genes in the TCGA-LIHC and ICGC-LIRI cohorts. (**D**). Kaplan-Meier survival curves for the TCGA-LIHC cohort. (**E**). Kaplan-Meier survival curves for the ICGC-LIRI cohort. (**F**). ROC curves showing the prediction performance of the nomogram in 1, 3, and 5-year OS in the TCGA-LIHC cohort. (**G**). ROC curves showing the prediction performance of the nomogram in 1, and 3-year OS in the ICGC-LIRI cohort

Following the differential expression analysis comparing subtypes, we selected 200 genes that exhibited specific upregulation in each subtype for further modeling studies (Table S1). These genes were subsequently subjected to univariate Cox analysis, which identified 145 PRGs (Figure S5). These 145 PRGs were then utilized in our machine learning computational framework to develop a CMLBS. Using our machine learning computational framework, we developed 101 different prediction models for the TCGA-LIHC cohort. Subsequently, we evaluated the performance of each model by calculating its C-index in the ICGC-LIRI cohort (Fig. 3A). Interestingly, the best model was the combination of StepCox [backward] and Enet [alpha = 0.6], which included 11 module genes and had the highest mean C-index (0.742), and this combination model exhibited a superior C-index in the validation cohort (Fig. 3A and C). The CMLBS score was calculated for each patient in all cohorts. Based on their own median values of CMLBS scores in the TCGA-LIHC and ICGC-LIRI cohorts, we stratified HCC patients into two groups: high-CMLBS and low-CMLBS, respectively (Table S2). High-CMLBS patients exhibited unfavorable clinical outcomes in both the TCGA-LIHC and ICGC-LIRI cohorts (Fig. 3D and E). The discriminatory capacity of CMLBS was assessed utilizing time-ROC analysis. In the TCGA-LIHC cohort, the area under the curve (AUC) values at 1, 3, and 5 years were 0.820, 0.760, and 0.745, respectively (Figs. 3F). For the ICGC-LIRI cohort, the AUC values at 1 and 3 years were 0.752 and 0.760, respectively (Figs. 3G).

### Evaluation of the CMLBS model

**Fig. 4:**
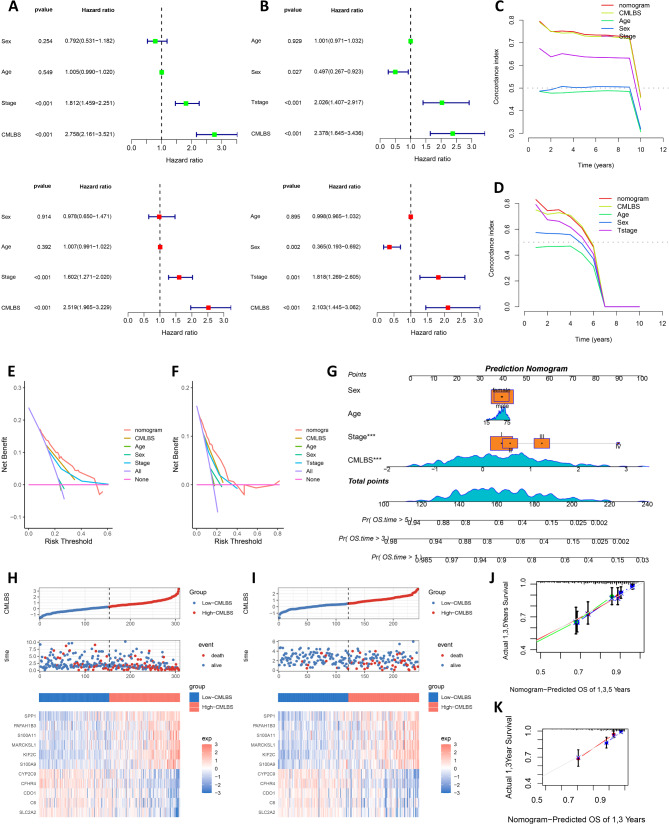
Evaluation of the CMLBS model. (**A**). Univariate and multivariate regression analysis of TCGA-LIHC cohort. (**B**). Univariate and multivariate regression analysis of ICGC-LIRI cohort. (**C**-**D**). The comparison of the C-index between the nomogram and other clinical characteristics in the TCGA-LIHC and ICGC-LIRI cohorts. (**E**-**F**). DCA analysis showing the net benefit by applying the nomogram and other clinical characteristics in the TCGA-LIHC and ICGC-LIRI cohorts. (**G**). Construction of the nomogram based on the CMLBS and clinical characteristics (including age, sex, and clinical stage) in the TCGA-LIHC cohort. (**H**). Heatmap of CMLBS distribution, patient survival, and expression profiles of 11 module genes that comprise CMLBS in the TCGA-LIHC cohort. (**I**). Heatmap of CMLBS distribution, patient survival, and expression profiles of 11 module genes that comprise CMLBS in the ICGC-LIRI cohort. (**J**). Calibration curve of the nomogram for 1, 3, and 5-year OS in the TCGA-LIHC cohort. (**K**). Calibration curve of the nomogram for 1 and 3-year OS in the ICGC-LIRI cohort

After controlling for potential confounders, including age, sex, AJCC staging, and T-staging, CMLBS remained an independent risk factor for HCC patients (Fig. 4A and B), and they were then integrated to construct a prognostic nomogram (Fig. 4G). Utilizing calibration curves, it was demonstrated that the accuracy of the nomogram in predicting 1-, 3-, and 5-year overall survival for HCC patients within the TCGA-LIHC cohort aligned with actual observations (Fig. 4J), and comparable results were also observed in the ICGC-LIRI cohort (Fig. 4K). The nomogram demonstrated superior predictive capabilities as evidenced by the C-index (Fig. 4C and D), while the DCA analysis further substantiated that its clinical benefit for HCC patients significantly surpassed that of CMLBS used independently (Fig. 4E and F). As illustrated in Fig. 4H-I, the CMLBS indicates the survival status of HCC patients and the expression levels of the 11 module genes. The SPP1, PAFAH1B3, S100A11, MARCKSL1, KIF2C, and S100A9 genes exhibited elevated expression levels in the high-CMLBS patients, while the CYP2C9, CFHR4, CDO1, C6, and SLC2A2 genes exhibited diminished expression levels in the high-CMLBS patients (Fig. 4H-I). Consequently, this observation provided additional evidence that the SPP1, PAFAH1B3, S100A11, MARCKSL1, KIF2C, and S100A9 genes function as detrimental factors, while the CYP2C9, CFHR4, CDO1, C6, and SLC2A2 genes function as favorable factors.

### Clinical correlations analysis of CMLBS model

**Fig. 5:**
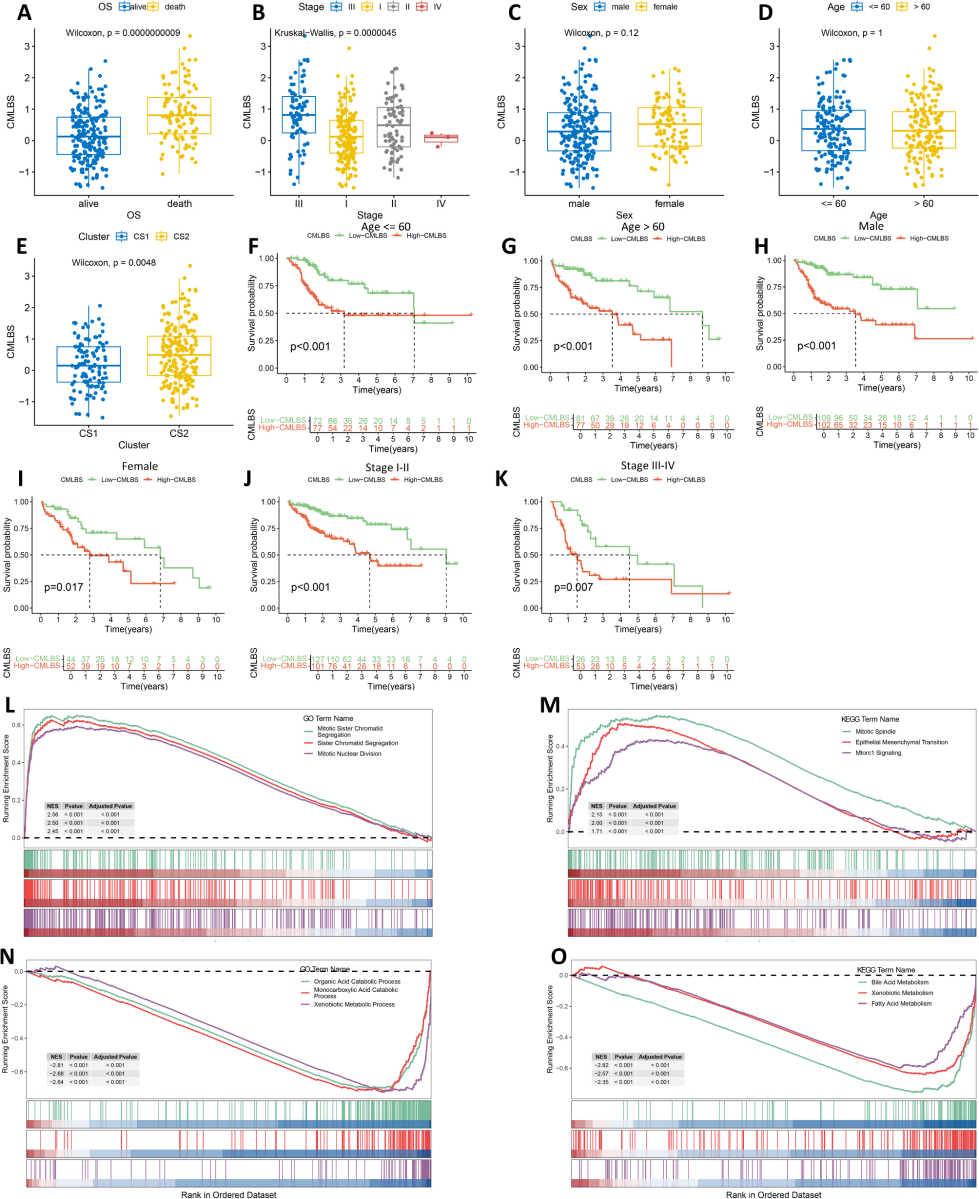
Clinical correlations analysis of CMLBS model. (**A**-**E**). Differences in CMLBS between groups with different clinical features. (**F**-**K**). OS KM curves for CMLBS in the two groups stratified by clinicopathologic factors. (**L**). The GO pathway enrichment analysis of the high-CMLBS group. (**M**). The KEGG pathway enrichment analysis of the high-CMLBS group. (**N**). The GO pathway enrichment analysis of the low-CMLBS group. (**O**). The KEGG pathway enrichment analysis of the low-CMLBS group

To validate the clinical relevance of CMLBS model, we investigated its variation across groups with distinct clinical characteristics. The findings indicated that patients with death, CS2, and stage III had higher CMLBS, implying that elevated CMLBS correlates with more advanced tumor progression (Fig. 5A and E). The analysis of survival data revealed that high-CMLBS patients had notably worse overall survival outcomes when compared to their low-CMLBS patients. This pattern was observed consistently across various clinical subgroups, including patients aged 60 and below, those above 60, both genders, patients diagnosed with stage I-II disease, and patients diagnosed with stage III-IV disease (Fig. 5F and K). Subsequently, we conducted GSEA analysis to investigate the pathways implicated in tumor development among low- and high-CMLBS patients. The findings indicated that the high-CMLBS patients exhibited a significant abundance in sister chromatid segregation, mitotic sister chromatid segregation, mitotic nuclear division, mitotic spindle, epithelial mesenchymal transition, and MTORC1 signaling (Fig. 5L and M). In addition, the low-CMLBS patients exhibited a significant abundance in monocarboxylic acid catabolic process, organic acid catabolic process, xenobiotic metabolic process, bile acid metabolism, xenobiotic metabolism, and fatty acid metabolism (Fig. 5N and O).

### Relationship between CMLBS model and immune microenvironment

**Fig. 6:**
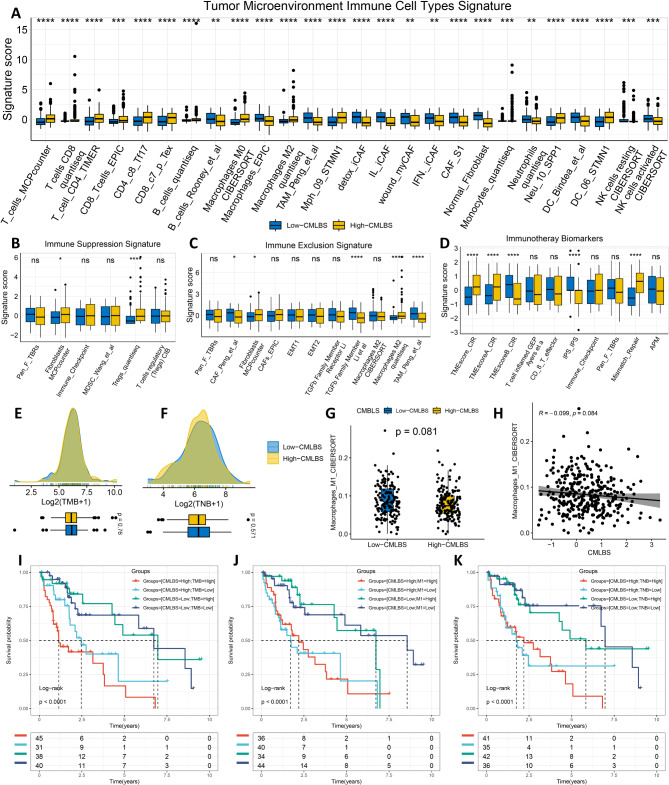
Relationship between CMLBS model and immune microenvironment. (**A**). The distribution of TME immune cell type signatures between high- and low-CMLBS patients. (**B**). The distribution of immune suppression signatures between high- and low-CMLBS patients. (**C**). The distribution of immune exclusion signatures between high- and low-CMLBS patients. (**D**). The distribution of immunotherapy biomarkers between high- and low-CMLBS patients. (**E**). The distribution of TMB between high- and low-CMLBS patients. (**F**). The distribution of TNB between high- and low-CMLBS patients. (**G**). The distribution of M1 macrophages between high- and low-CMLBS patients. (**H**). The relationship between CMLBS and M1 macrophages. (**I**-**K**). Survival analysis combined CMLBS with TMB, TNB, and M1 macrophages

Utilizing the IOBR package, we performed an extensive evaluation of the HCC tumor microenvironment (TME) and noticed that infiltration levels of B cells, T cells, macrophages, and dendritic cells were considerably elevated in high-CMLBS patients than in low-CMLBS patients, indicating an enhanced immune state (Fig. 6A, Figure S6A-S6F, Figure S8 and Figure S9). The results indicated that high-CMLBS group is more prone to be categorized as “hot tumors”. Conversely, low-CMLBS patients exhibited an enrichment of molecular indicators associated with immunosuppression and exclusion, such as TAM, demonstrating an immunosuppressive environment (Fig. 6B and C). This finding suggests that the low-CMLBS group exhibits a higher propensity for classification as “cold tumors”. Consistent with our predictions, previously identified signatures correlated with improved immunotherapy outcomes were also notably abundant in the high-CMLBS group (Fig. 6D). We observed no statistically significant differences in the enrichment of TMB, TNB and M1 macrophages in the two CMLBS groups (Fig. 6E and H). The analysis of survival data revealed that CMLBS could function as an efficacious supplementary factor alongside TMB, TNB, and M1 macrophages in differentiating outcomes for HCC patients. Patients with HCC who presented a combination of lower CMLBS and elevated levels of TMB, TNB, or M1 macrophage infiltration were associated with more favorable survival prognoses (Fig. 6I and K). Furthermore, SPP1, S100A9, and S100A11 genes exhibited strong positive associations with the majority of tumor-infiltrating immune cells, while PAFAH1B3, MARCKSL1, KIF2C, CYP2C9, CFHR4, CDO1, C6, and SLC2A2 genes exhibited significant negative associations with most tumor-infiltrating immune cells (Figure S6G and Figure S7).

### Predictive efficacy of immunotherapy response for the CMLBS model

**Fig. 7:**
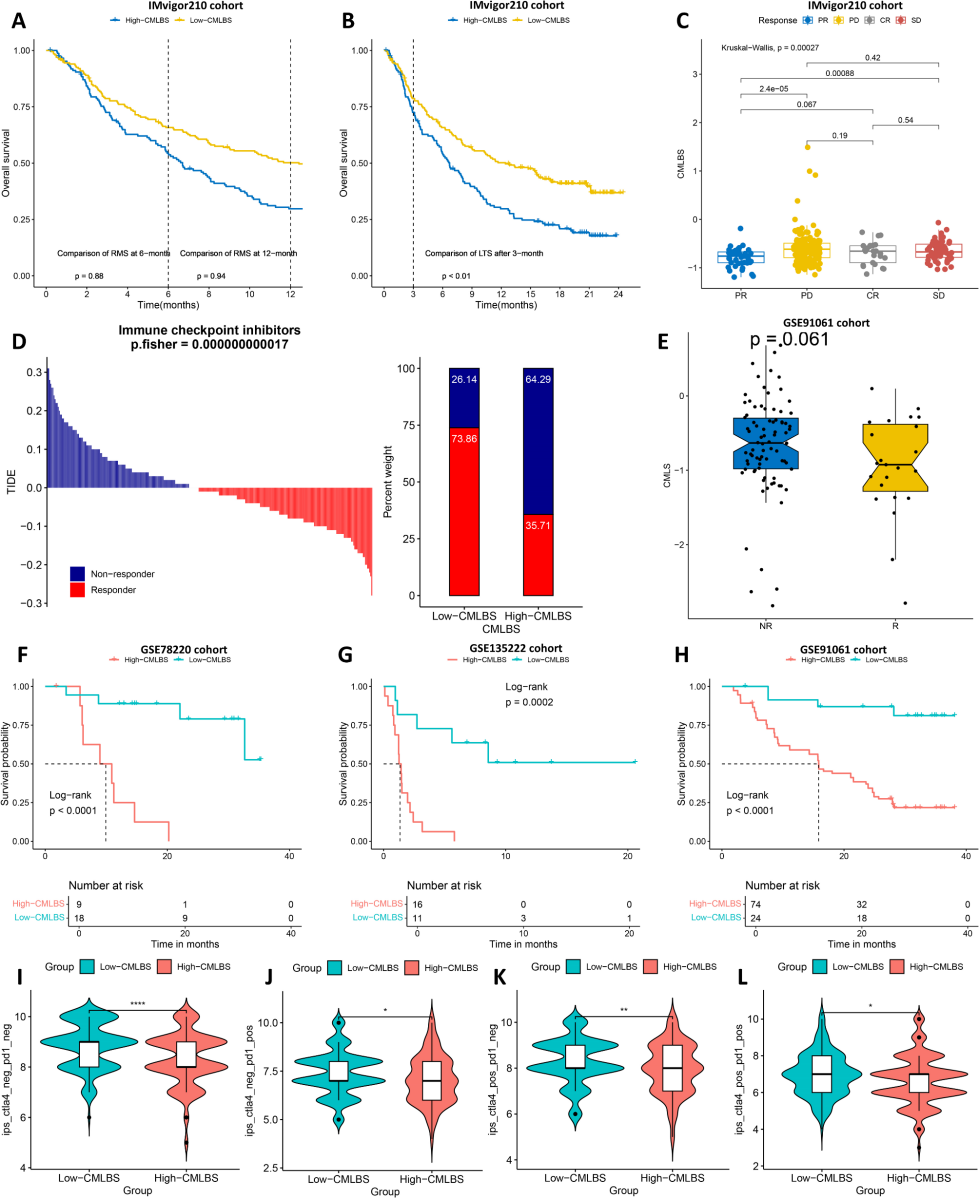
Predictive efficacy of immunotherapy response for the CMLBS model. (**A**). The restricted mean survival (RMS) time difference by 6 months and 12 months after treatment between high- and low-CMLBS groups. (**B**). The long-term survival (LTS) difference after 3 months of treatment between high- and low-CMLBS groups. (**C**). The distribution of CMLBS in different immunotherapy response groups. (**D**) The TIDE algorithm predicts response to immunotherapy between high- and low-CMLBS groups. (**E**). Distribution of CMLBS in different immunotherapy response groups of GSE91061. (**F**). Kaplan-Meier survival curves for the GSE78220 cohort. (**G**). Kaplan-Meier survival curves for the GSE135222 cohort. (**H**). Kaplan-Meier survival curves for the GSE91061 cohort. (**I**-**L**). Comparisons of the IPS between high- and low-CMLBS groups

To evaluate the delayed clinical impact of immunotherapy, we compared the restricted mean survival (RMS) between the two patient groups at 6 and 12 months, and concurrently assessed the difference in long-term survival among patients following 3 months of treatment (*P* < 0.01; Fig. 7A and B). The superior prognostic outcomes observed in low-CMLBS patients suggested that immunotherapy demonstrates enhanced efficacy for this group. Analysis of the CMLBS distribution demonstrated that the responder group (complete response [CR]/partial response [PR]) exhibited a considerably lower CMLBS score in comparison to the non-responder group (progressive disease [PD]/stable disease [SD]) (*P* < 0.05; Fig. 7C). In addition, the TIDE algorithm was employed to assess the immunotherapy response of patients, demonstrating enhanced responsiveness among the low-CMLBS patients (*P* = 0.000000000017; Fig. 7D). Following this, we confirmed our findings in several immunotherapy validation cohorts that included prognostic data. The low-CMLBS patients exhibited significantly improved prognostic outcomes in GSE91061cohort (*P* < 0.0001; Fig. 7E and H), GSE78220 cohort (*P* < 0.0001; Fig. 7F), and GSE135222 cohort (*P* = 0.0002; Fig. 7G). The results of the immunophenoscore indicated that immune checkpoint inhibitor therapy demonstrated greater therapeutic efficacy in low-CMLBS patients (Figs. 7I-2L). Employing ssGSEA methodology, we quantified immune-related pathway scores, demonstrating markedly elevated Type I IFN Response and Type II IFN Response activity in low-CMLBS patients (**Figure S10A**). Notably, 11 module genes in CMLBS, excluding SLC2A2, CYP2C9, CFHR4, CDO1 and C6 genes, exhibited positive associations with numerous immune checkpoints (**Figure S10B**). With the exception of IDO2 gene, the majority of immune checkpoints exhibited elevated expression in high-CMLBS patients (**Figure S10C**).

### Chemotherapeutic sensitivity prediction

**Fig. 8:**
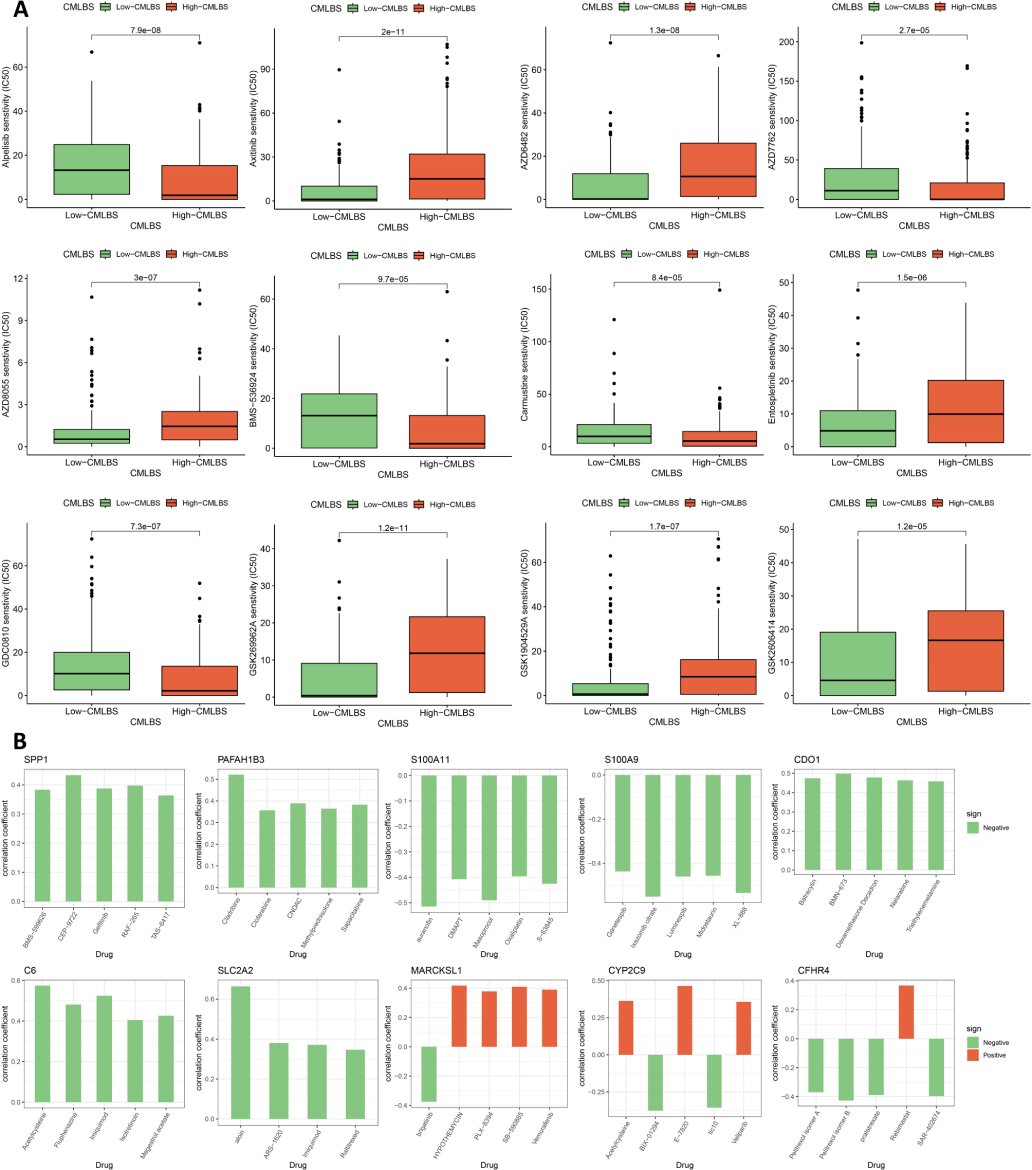
Chemotherapeutic sensitivity prediction. (**A**). Differences in response to commonly used chemotherapeutic drugs between the high- and low-CMLBS groups. (**B**). Correlation between 11 module genes constructed CMLBS and drug sensitivity

To further investigate the clinical role of module genes in the precision treatment of HCC patients, we evaluated the efficacy of commonly used chemotherapeutic drugs across various CMLBS groups. We found that the high-CMLBS patients may exhibit increased sensitivity to Alpelisib, AZD7762, BMS-536,924, Carmustine, and GDC0810, whereas they may demonstrate reduced sensitivity to Axitinib, AZD6482, AZD8055, Entospletinib, GSK269962A, GSK1904529A, and GSK2606414 (Fig. 8A). In addition, we observed that the expression levels of the SPP1, PAFAH1B3, S100A11, S100A9, CDO1, C6, and SLC2A2 genes exhibited a negative correlation with the majority of the conventional chemotherapeutic drugs available in the CellMiner database, suggesting that increased expression of these genes is associated with enhanced resistance to the aforementioned drugs (Fig. 8B).

## Discussion

The high morbidity and mortality of HCC impose a substantial economic burden on patients’ families and society [18]. The majority of HCC patients are detected at advanced stages and experience poor therapeutic outcomes, whereas early-stage patients exhibit the most favorable prognosis following radical treatment [4]. Consequently, early detection of HCC can significantly improve patients’ prognosis and reduce the overall disease burden. However, significant challenges persist in effectively determining the prognosis of HCC patients, accurately assessing the severity of disease in HCC patients, and precisely identifying HCC patients who will benefit from immunotherapy. Molecular typing of tumors more accurately reflects differences in molecular characteristics within neoplasms and plays a crucial role in the personalized management of patients in clinical practice. Currently, investigations into molecular subtyping of tumors predominantly utilize data from a single omics source, such as genomics, transcriptomics, proteomics, and epigenomics [15, 19–21]. However, a singular genomics study can only elucidate tumor-related molecular information at a single level. Therefore, the integrated analysis of multi-omics data can deeply analyze the heterogeneity of neoplasms and integrate the data from multiple dimensions to establish more precise molecular typing of tumors. In this study, we employed the latest 10 different clustering algorithms to identify two molecular subtypes of HCC by incorporating data from mRNA, lncRNA, miRNA, epigenomic DNA methylation expression profiles, and genomic mutations, which may offer potential value for the precise stratified treatment of HCC patients.

To address the high dimensionality and volume of multi-omics data, machine learning algorithms can be utilized to integrate and analyze high-throughput multi-omics data to discover novel biomarkers [22–24]. To elucidate the molecular distinctions among prognostic subtypes and enhance the clinical utility, we employed a diverse set of 101 combinations derived from 10 machine learning algorithms to develop a CMLBS model to mitigate the limitations typically associated with algorithm selection. Currently, the combination of multi-omics data and machine learning encounters many challenges, among which overfitting represents a critical concern that warrants careful consideration during model development, which is mainly manifested in the fact that the model exhibits excellent performance on the training dataset, while exhibiting poor performance when evaluated on the testing dataset [22]. To mitigate the issues arising from overfitting in the training cohort, we employed the mean C-index of both training and validation cohorts as our ranking criterion, which identified the optimal CMLBS model as a combination of StepCox [backward] and Enet [alpha = 0.6].

The optimal CMLBS model comprises 11 module genes: PAFAH1B3, S100A11, CFHR4, SLC2A2, CYP2C9, SPP1, C6, CDO1, S100A9, MARCKSL1, and KIF2C. While these 11 module genes exhibit crucial functions in tumor initiation and advancement, they demonstrate no mutual correlation. PAFAH1B3 is a critical metabolic enzyme in driving triple-negative breast carcinogenesis, and targeted suppression of PAFAH1B3 results in the upregulation of multiple oncogenic signaling pathways, which contributes to the inhibition of proliferation and invasive capacity of cancer cells, and may potentially serve as a novel therapeutic target [25]. S100A11 was observed to be highly expressed in pancreatic ductal adenocarcinoma (PDAC) tissues and demonstrated a significant association with poorer prognosis and disease progression [26]. It was found that CFHR4 exhibits a significant association with immune cell infiltration and may be considered a potential therapeutic target for enhancing HCC prognosis [27]. SLC2A2 was identified as a new prognostic factor in HCC patients [28]. Genetic polymorphisms in CYP2C9 have been demonstrated to exhibit an association with colorectal cancer risk [29]. It was found that SPP1 exhibits high expression levels in esophageal carcinoma tissues and plays a significant role in the development, prognosis, and diagnosis of esophageal carcinoma and the SPP1/JAK2/STAT3 axis was found to be crucial in the progression of esophageal carcinoma and in conferring radiotherapy tolerance [30]. It was found that C6 exhibits low expression in esophageal carcinoma tissues, potentially attributable to alterations in gene expression regulation [31]. CDO1 alters cytosine metabolism, leading to the production of reactive oxygen species (ROS) and a reduction in cell viability and growth, and is considered to be a key tumor suppressor gene as well as a molecular marker of chemoresistance in cancer cells [32]. S100A9 has been identified as a crucial driver in the progression of HCC following transarterial chemoembolization and is implicated in multiple growth and metastatic processes in HCC, particularly influencing mitochondrial function [33]. In addition, MARCKSL1 has been implicated in the progression of various malignancies, including breast, lung, and esophageal squamous cell carcinoma [34–36]. KIF2C exhibits elevated expression in the majority of neoplasms and demonstrates correlations with clinical prognosis, oncogenic signature gene sets, myeloid immune cell infiltration, immune scores, immune checkpoints, microsatellite instability, tumor mutational burden, and clinical outcomes across various malignancies [37].

Utilizing the IOBR package, we executed an all-inclusive quantification of the tumor immune microenvironment in the low- and high-CMLBS groups. We found that various immune cells exhibited significant activation and demonstrated an increased propensity for developing a hot tumor phenotype [38]. In addition, the results of TIDE showed greater responsiveness to immunotherapy in the low-CMLBS patients. Survival analyses additionally demonstrated that the low-CMLBS patients exhibited a more favorable prognosis, a finding that was consistently observed across multiple immunotherapy cohorts, indicating that CMLBS may serve as a valuable tool for identifying HCC patients that derive benefit from immunotherapy. Encouragingly, we observed that the high-CMLBS patients may exhibit increased sensitivity to Alpelisib, AZD7762, BMS-536,924, Carmustine, and GDC0810, whereas they may demonstrate reduced sensitivity to Axitinib, AZD6482, AZD8055, Entospletinib, GSK269962A, GSK1904529A, and GSK2606414, suggesting that CMLBS may contribute to the selection of chemotherapeutic agents for HCC patients. Despite the lack of statistical difference in age and gender within the nomogram in our study, numerous studies have evaluated the recent incidence and mortality of HCC, as well as temporal trends. Examining the differences between gender, age, and ethnic race facilitates the identification of high-risk groups, which in turn guides early detection, intervention, and treatment, ultimately improving patient prognosis. While we acknowledge that age and gender are generally considered important factors in HCC prognosis, our specific datasets and patients’ cohort may have unique characteristics that contribute to the observed lack of statistical significance.

Nevertheless, it is important to acknowledge that this study has certain limitations: 1). The conclusions of this study primarily relied on HCC cohort analyses and computational simulations from public databases, which may be subject to bias due to variations in database platforms. 2). In this study, the CMLBS model was not compared with existing prognostic models for HCC to elucidate its superior predictive performance, as the collection of information on existing prognostic models for HCC is both complex and extensive. 3). The clinical utility of the CMLBS model requires further validation through large-scale, multicenter clinical cohorts. 4). The specific mechanisms underlying the relationship between CMLBS model genes and HCC development necessitate further vitro and vivo experimental studies.

## Conclusion

Through multi-omics consensus clustering, this research uncovered two distinct molecular subtypes of HCC, revealing notable variations in prognosis between these subtypes, and potentially enhancing the molecular classification of HCC. In addition, using our machine learning computational framework, we developed a CMLBS that effectively predicted patient outcomes in multiple cohorts while also demonstrating strong correlations with responses to immunotherapy, indicating that CMLBS may serve as a valuable tool for HCC patients.

## Materials and methods

### Datasets collection and processing

We initially acquired multi-omics data of the TCGA-LIHC cohort from the Cancer Genome Atlas (TCGA) database (https://portal.gdc.cancer.gov), encompassing HCC patients with comprehensive transcriptome profiles, epigenomic DNA methylation profiles, somatic mutations, and clinical data. The expression spectrums of mRNA and lncRNA were acquired utilizing the TCGAbiolinks package [39]. The identification of mature miRNA of TCGA-LIHC cohort was recognized utilizing the miRBaseVersions.db package. The data of somatic mutations was similarly acquired through TCGAbiolinks [39] and subsequently treated using the maftools package [40]. The epigenomic DNA methylation profiles and clinical data were procured from UCSC XENA (https://xenabrowser.net/). Subsequently, we acquired the comprehensive data of ICGC-LIRI cohort from the International Cancer Genome Consortium (ICGC) database, and acquired the comprehensive data of GSE14520 cohort, GSE76427 cohort, GSE78220 cohort, GSE135222 cohort, and GSE91061 cohort from the Gene Expression Omnibus (GEO) databases. Finally, we obtained the IMvigor210 dataset by employing the “IMvigor210CoreBiologies” R-package [41], which encompasses clinical data pertaining to the utilization of anti-PD-L1 monoclonal antibody (atezolizumab) for urothelial carcinoma treatment [42]. The RNA-seq data was transformed to transcripts per kilobase million (TPM), while all microarray data underwent deduplication and standardization processes. Among these cohorts, TCGA-LIHC was designated as the training cohort, while the remaining cohorts were considered validating cohorts.

### Integrative clustering based on multi-omics profiles

To conduct a thorough analysis, we initially aligned the omics data from the five dimensions using the sample ID, encompassing a total of 326 samples. A log2(TPM + 1) transformation was applied to the TPM expression data. For the epigenomic DNA methylation profiles, we extracted the probes of promoter CpG islands. In the construction of the gene mutation matrix, a gene was designated as mutated if it exhibited any nonsynonymous variations from the following categories: deletion or frameshift insertion, in-frame deletion or insertion, splice site or nonstop mutation or missense, or nonsense or translation start site mutation. The MOVICS package [43] was implemented for gene feature selection to boost model fit and streamline the clustering process. Particularly, we selected the 1,500 genes exhibiting the highest variability across mRNA, lncRNA, miRNA, and methylation categories, based on their median absolute deviation scores. For gene mutation data, we initially selected the 5,000 genes with the highest mutation frequencies and subsequently identified the top 5% of genes that showed the most frequent mutations. We utilized the MOVICS package [43] to determine the optimal number of clusters by calculating the clustering prediction index (CPI), gap statistics, and Silhouette score. Finally, we used 10 distinct clustering algorithms (CIMLR, PINSPlus, COCA, ConsensusClustering, IntNMF, iClusterBayes, MoCluster, LRAcluster, NEMO, and SNF) to conduct clustering analysis in the TCGA-LIHC cohort. We initially validated the clustering results utilizing subtype-specific biomarkers in the validation cohorts to evaluate the robustness of the identified subtypes. Subsequently, we then proceeded to analyze the concordance between identified subtypes and the NTP and PAM classifiers [43]. Using established protocols, the DNA methylation of tumor-infiltrating lymphocytes (MeTIL) was determined for the identified subtypes [44].

### Development of a consensus machine learning-based prognostic signature

To develop CMLBS with enhanced accuracy and broad applicability, we incorporated 10 distinct machine learning algorithms (stepwise Cox, CoxBoost, survival support vector machine (survival-SVM), Ridge, generalized boosted regression models (GBMs), elastic net (Enet), supervised principal components (SuperPC), Lasso, partial least Cox (plsRcox), and survival forest (RSF)) and 101 different algorithm combinations. In implementing the CoxBoost model, we initially utilized the “optimCoxBoostPenalty” function to identify the ideal penalty value. Subsequently, we integrated this with cross-validation, executing a 10-fold cross-validation process on the CoxBoost model to determine the optimal number of boosting steps. Lastly, we employed the “CoxBoost” function to construct the final model. Using the survival package, we conducted a stepwise Cox analysis. The Akaike information criterion (AIC) was employed to evaluate the statistical model’s complexity. We investigated all potential combinations for the direction parameter, encompassing “both,” “backward,” and “forward” options. The glmnet package and its “cv.glmnet” function were employed to develop Lasso, Ridge, and Enet models. Cross-validation with 10 folds was utilized to determine the optimal lambda regularization parameter. The alpha tradeoff parameter was adjusted within the range of 0 to 1, using increments of 0.1. Lasso is implemented when alpha equals 1, while Ridge is applied when alpha is 0. For alpha values between 0 and 1, Enet is utilized. The survival-SVM model was developed through the application of the “survivalsvm” function, which is provided by the survivalsvm package. This particular function is designed to conduct support vector analysis on datasets that contain survival information. The implementation of the GBM model utilized the gbm package. A GBM was fitted using the “gbm” function, which incorporated 10-fold cross-validation. The implementation of the SuperPC model utilized the superpc package, an extension of Principal Component Analysis (PCA). Additionally, the “superpc.cv” function was employed with 10-fold cross-validation. The plsRcox package’s “cv.plsRcox” function was utilized for the plsRcox model. Regarding the RSF model, the randomForestSRC package was implemented, specifically employing the “rfsrc” function with two primary parameters: “ntree” and “nodesize.” In random forest models, “ntree” indicates the total number of trees, while “nodesize” specifies the smallest allowable size for terminal nodes. For this research, we configured “ntree” to 1,000 and set “nodesize” to 5. The process for generating CMLBS encompassed the following step: (a) Utilizing univariate Cox regression to identify prognosis-related genes (PRGs) in the TCGA-LIHC cohort; (b) Subsequently, 101 distinct algorithm combinations were utilized to develop the most optimal CMLBS, which exhibited the highest C-index performance within the TCGA-LIHC cohort; (c) All models were subjected to evaluation in the ICGC-LIRI cohort; (d) The C-index was computed in the validation cohort for each model. The model demonstrating the highest mean C-index was identified as the most optimal; (e) The weight coefficients for each gene from the most model, subsequently recalculated using multivariate Cox analysis; (f) Both univariate and multivariate Cox analyses were executed to identify whether the CMLBS could function as an independent predictor for HCC patients in all cohorts.

### Construction and validation of the prognostic nomogram

To begin, we applied the resultant model to compute CMLBS for each patient in the TCGA-LIHC, ICGC-LIRI, and multiple immunotherapy cohorts. Based on their own median values of CMLBS scores in the TCGA-LIHC and ICGC-LIRI cohorts, we stratified HCC patients into two groups: high-CMLBS and low-CMLBS, respectively. Subsequently, we employed the surv_cutpoint function from the survminer package to analyze the relationship between CMLBS and survival outcomes in the immunotherapy cohort. This function identifies the cutoff point that optimally distinguishes between survival curves, thereby ensuring that the division of high- and low-CMLBS scores is based on statistically significant differences in survival. We assessed the prognostic value of CMLBS using Kaplan-Meier survival analysis. Subsequently, we employed multivariate and stepwise Cox regression analyses to develop predictive nomogram for HCC patients. Following this, the accuracy was evaluated using time-ROC and calibration curves, while the decision curve analysis (DCA) was employed to assess the clinical benefit for HCC patients. Ultimately, correlation analysis and stratified analysis were employed to explore the association between CMLBS and clinical features among HCC patients.

### Gene set enrichment analysis

To examine the core biological processes, Gene Set Enrichment Analysis (GSEA) was executed using the R packages “clusterProfiler” [45], “enrichplot”, and “GseaVis”. The selection criteria required a|NES| > 1 and a *P* < 0.05.

### Immunogenomic landscape analysis

Utilizing the IOBR package [46], we gathered numerous previously published signatures associated with TME cell types, responses to immunotherapy, immune exclusion, and immune suppression. We then employed a standardized method to compute enrichment scores for each patient, enabling a comprehensive analysis of immunological distinctions across the two groups. A comparative analysis was conducted to examine the dispersion of TNB, TMB, and M1 macrophages across the two CMLBS groups. Furthermore, HCC patients underwent reclassification based on CMLBS.

### Identification of the immunotherapy efficacy

To assess the immunotherapy response, we initially examined the patients’ survival rates following delayed response to immunotherapy. We integrated data from The Cancer Immunome Atlas platform (TCIA, https://tcia.at/) [47] and the Tumor Immune Dysfunction and Rejection platform (TIDE, http://tide.dfci.harvard.edu/) [48] to forecast immunotherapy outcomes. Our findings were subsequently validated using the IMvigor210 [42], GSE135222 [49], GSE78220 [50], and GSE91061 [51] cohorts.

### Comparisons of chemotherapeutic drug sensitivity

Statistically significant differences were ascertained by comparing chemotherapeutic drug sensitivities between the two groups, and the “oncoPredict” [52] R software package was employed to compute half-maximum inhibitory concentration (IC50) values for commonly used anticancer drugs. Moreover, we executed a thorough analysis of the expression levels of the module genes comprising the CMLBS and drug sensitivity, utilizing the CellMiner database [53].

### Statistical analysis

Survival curves were analyzed utilizing the Kaplan-Meier method. To compare multiple groups or two groups, the Wilcoxon test or the Kruskal-Wallis’s test was applied, respectively. Spearman’s correlation analysis was implemented to evaluate correlations. R software version 4.4.0 was employed for conducting statistical analyses. Statistical discrepancies were determined at *P* ≤ 0.05.

## Electronic supplementary material

Below is the link to the electronic supplementary material.

Supplementary Material 1

Supplementary Material 2

## Data Availability

No datasets were generated or analysed during the current study.
