# Supplementary material for: Integrated multi-omics analysis and machine learning refine molecular subtypes and clinical outcome for hepatocellular carcinoma
Source: Hereditas. 2025 Apr 12;162:61. doi: 10.1186/s41065-025-00431-6 (PMC11992824; doi:10.1186/s41065-025-00431-6)
Supplement: Supplementary file 1 — Supplementary Material 1 [file 41065_2025_431_MOESM1_ESM.docx]

**Supplementary Figures**

**Article Title**: Integrated multi-omics analysis and machine learning refine molecular subtypes and clinical outcome for hepatocellular carcinoma


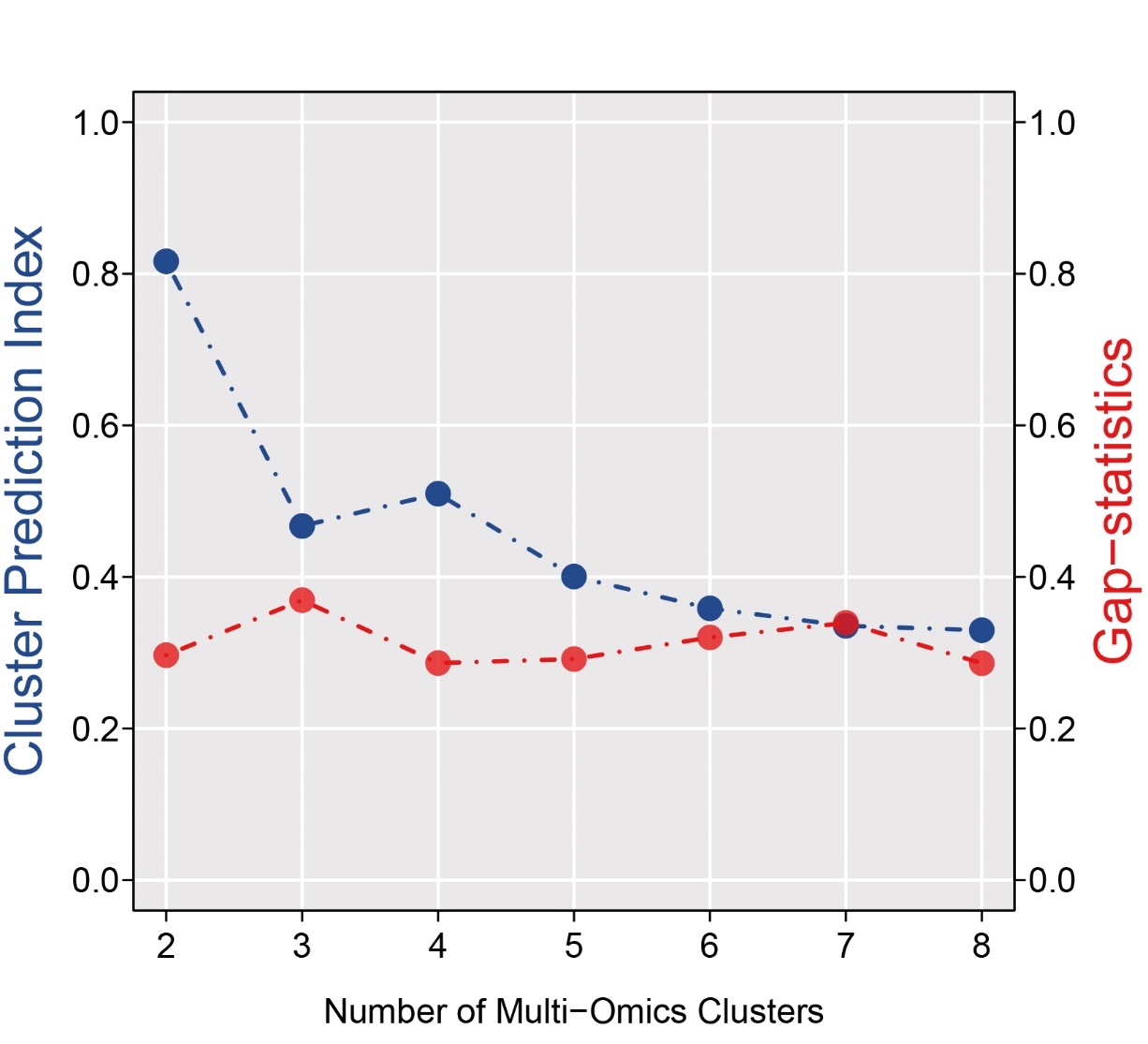


**Figure S1.** The cluster prediction index and gap statistical analysis of the multi-omics clusters.


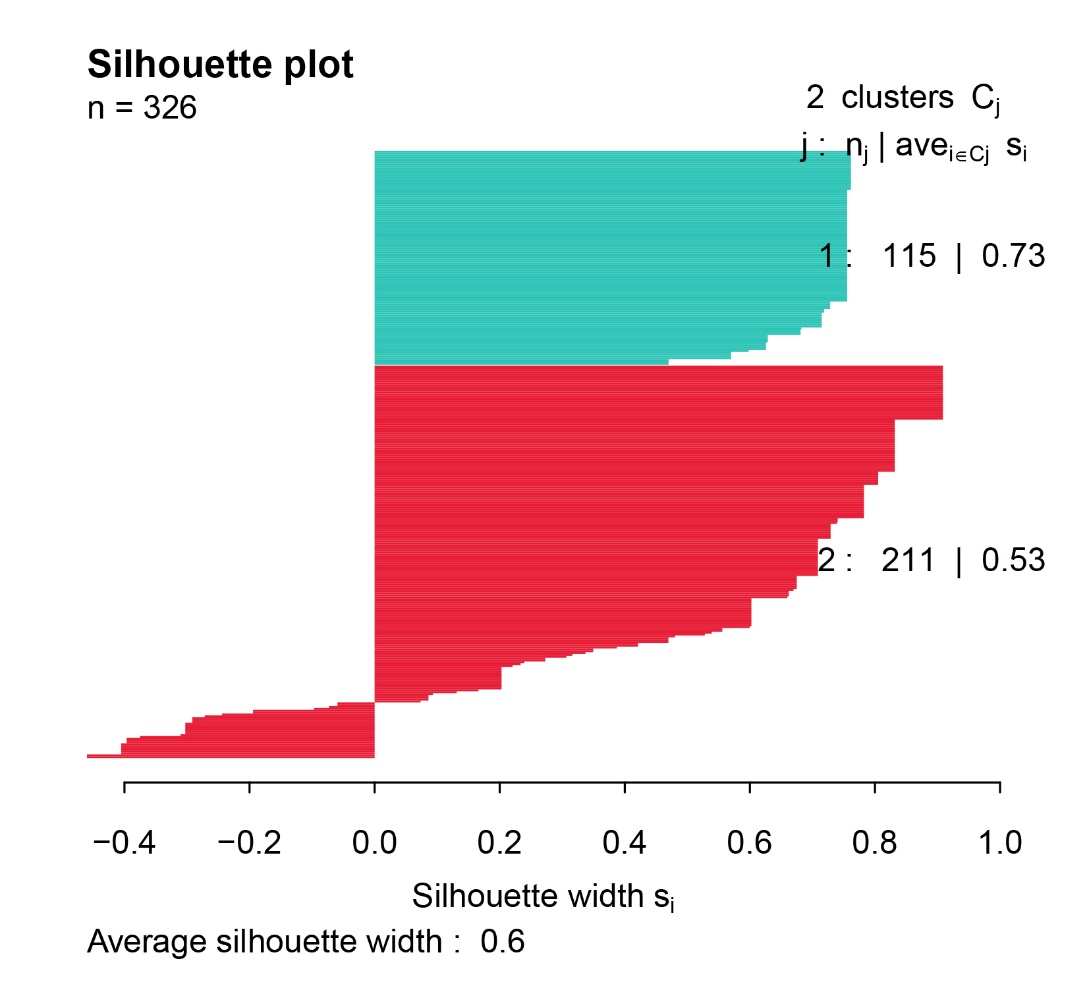


**Figure S2.** The sample similarity of each subgroup was assessed by calculating the Silhoutte score.


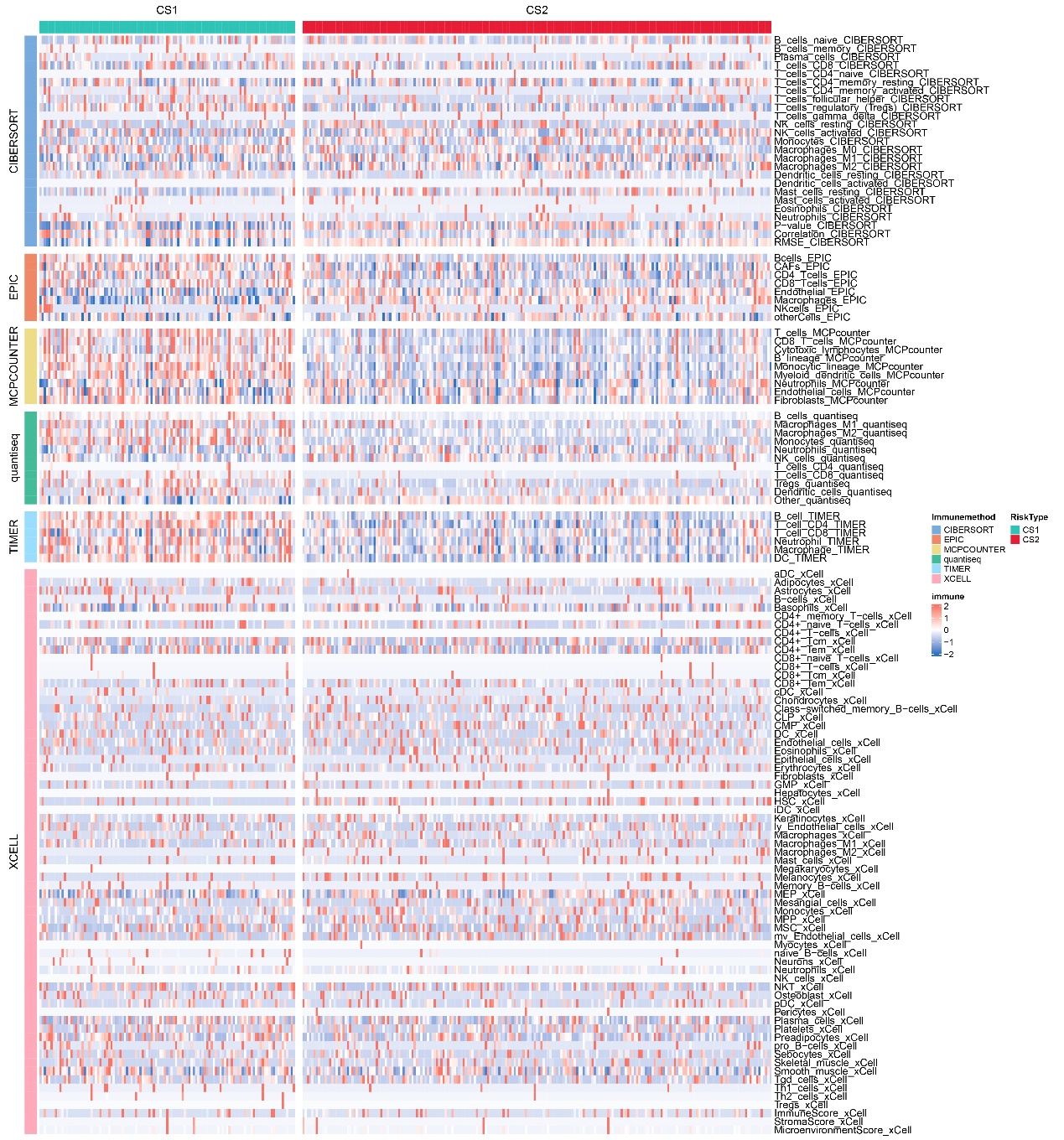


**Figure S3.** Six other algorithms, including the MCP-counter algorithm, EPIC algorithm, xCell algorithm, CIBERSORT algorithm, quanTIseq algorithm, and TIMER algorithm, further verified the stability and robustness of the ssGSEA results for two clusters.


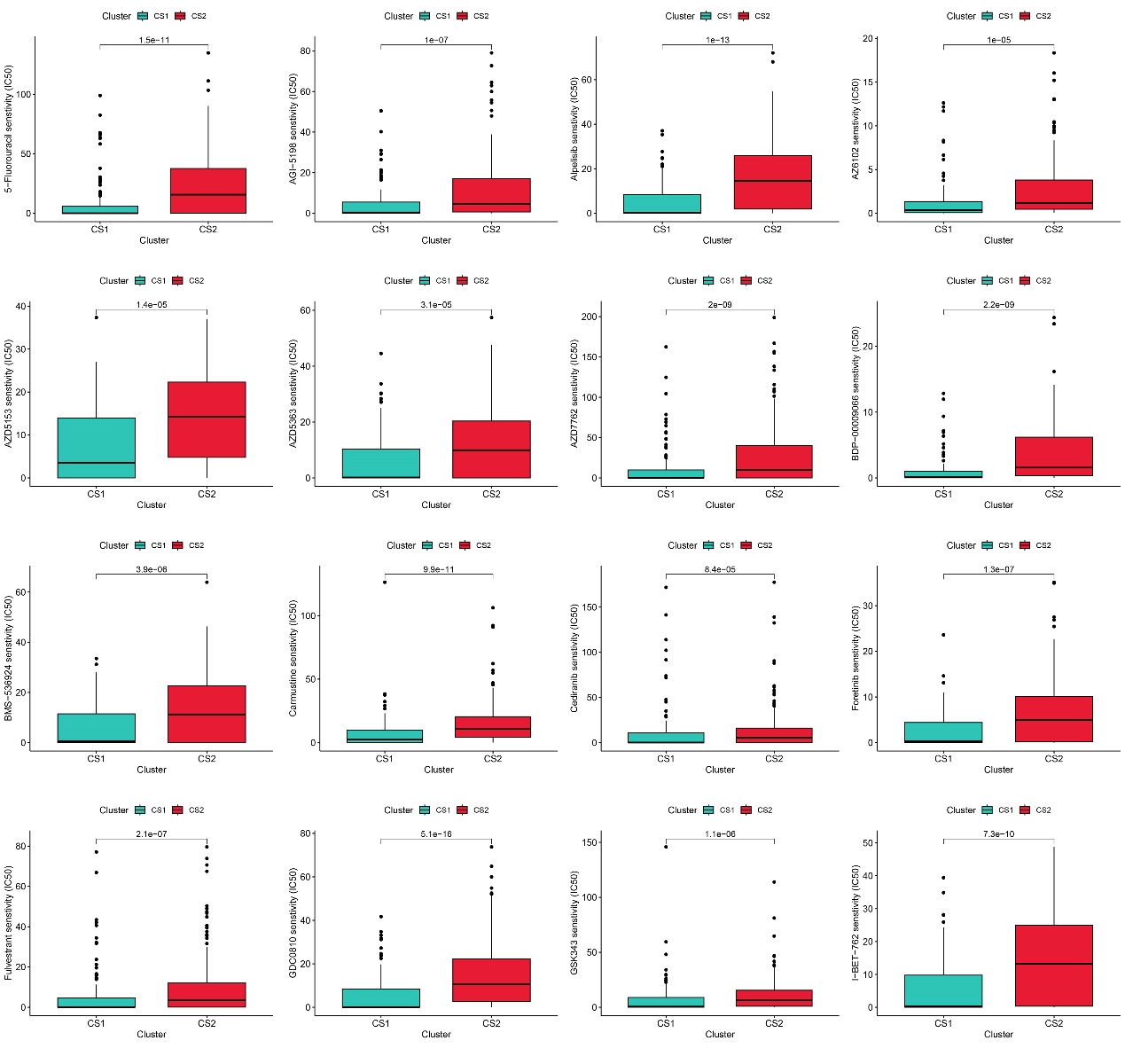


**Figure S4.** Differences in response to commonly used chemotherapeutic drugs for two clusters.


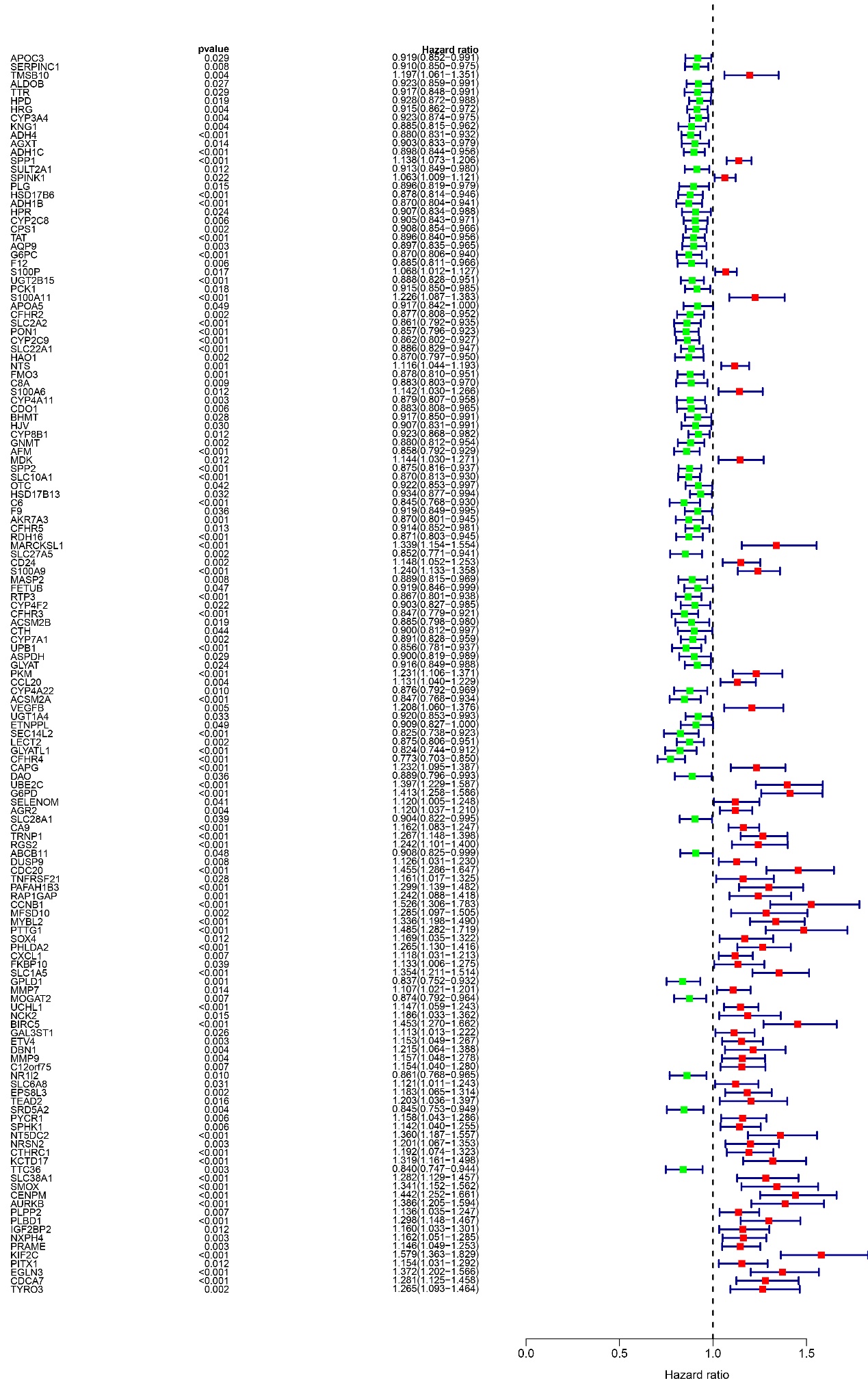


**Figure S5.** Univariate Cox analysis identified prognosis-related genes in the TCGA-LIHC cohort.


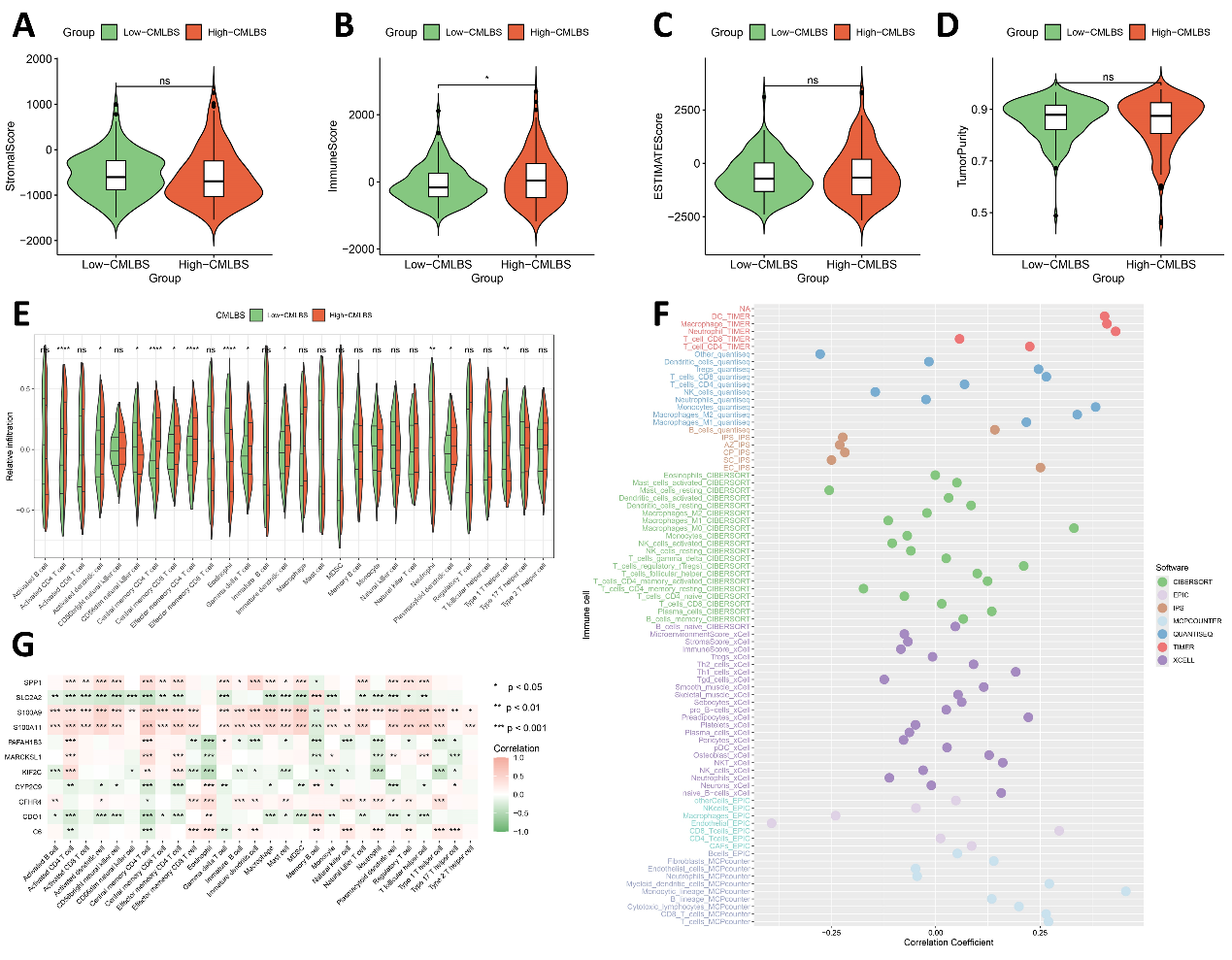


**Figure S6.** Identification of the immune landscape. (A-D). Stroma score, Immune score, ESITIMATE score, and Tumor purity in the two CMLBS groups. (E). ssGSEA score of immune cell infiltration in the two CMLBS groups. (F). Correlation between immune scores and immune cells assessed by different software. (G). The correlation between each key molecule and each TME infiltration cell type.


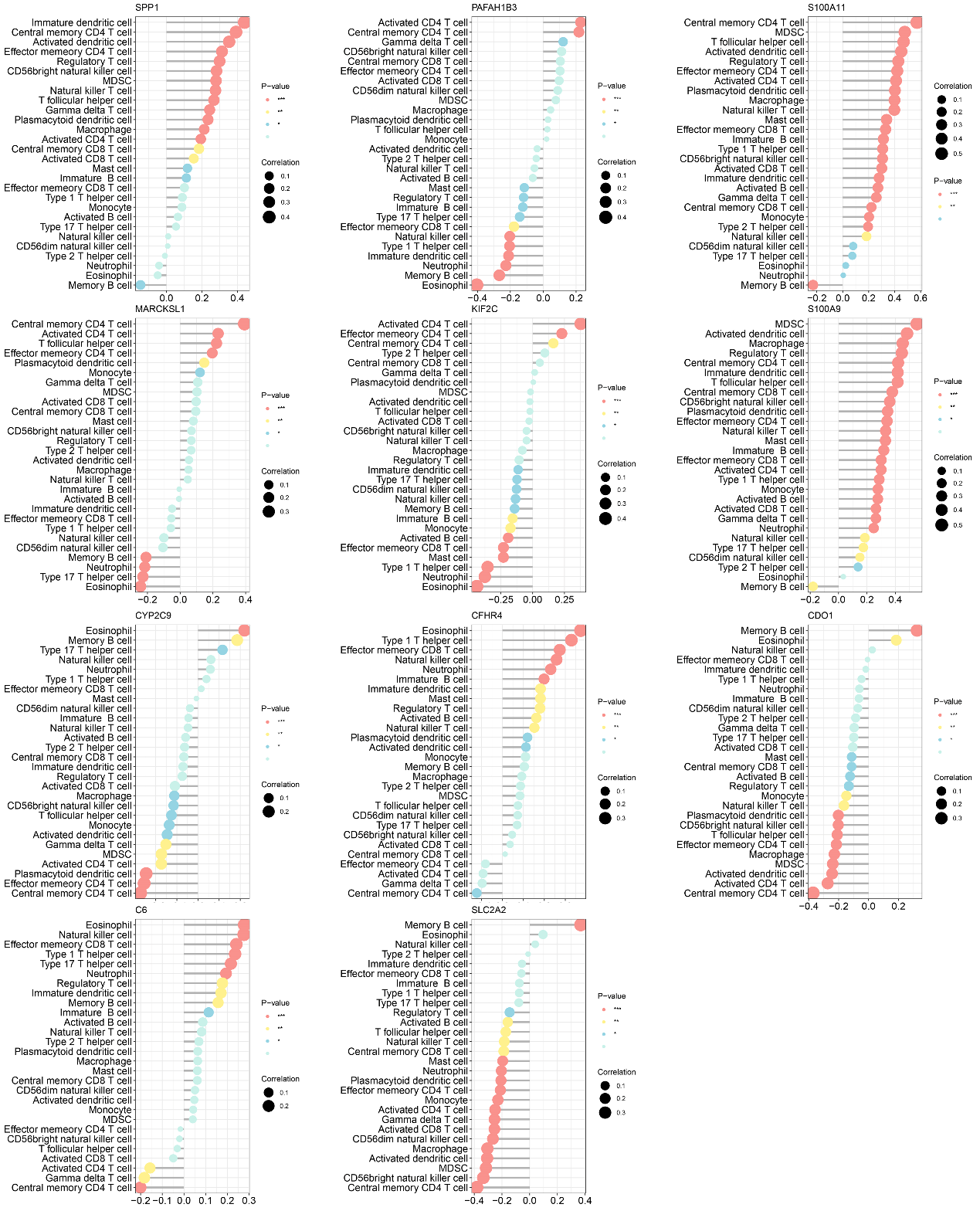


**Figure S7**. The correlation between each key molecule and each TME infiltration cell type.


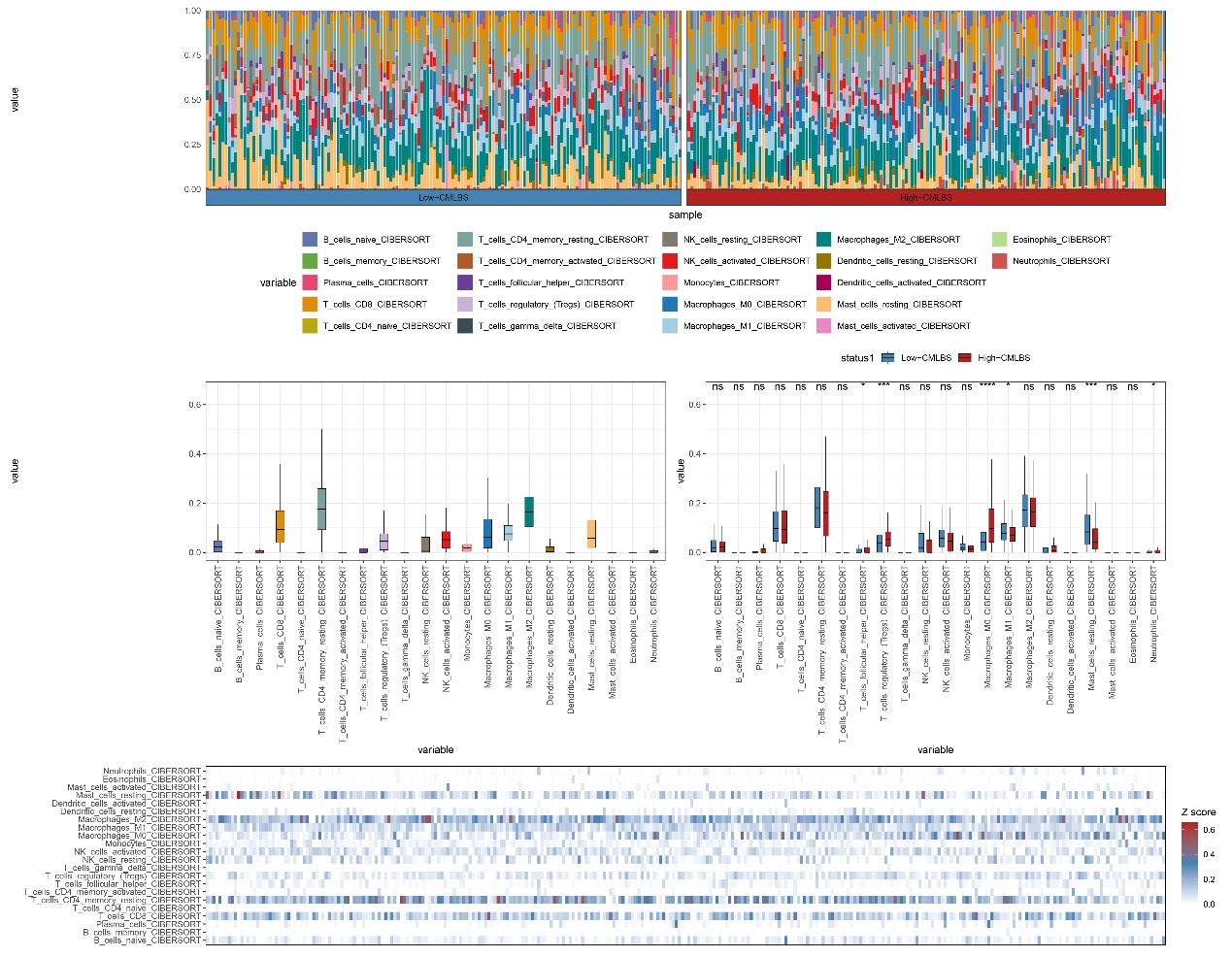


**Figure S8**. CIBERSORT score of immune cell infiltration between the two CMLBS groups.


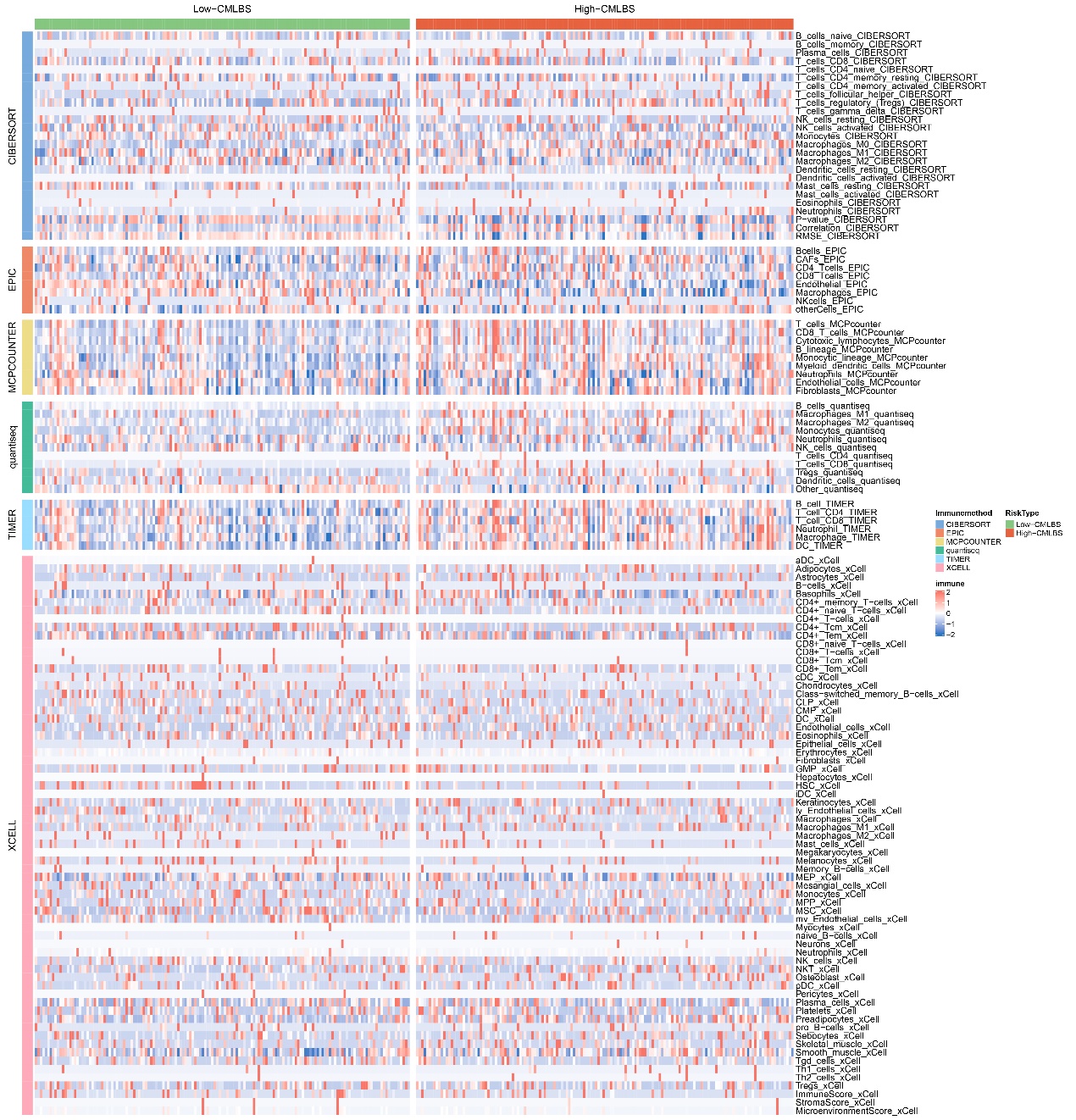


**Figure S9**. Six other algorithms, including the MCP-counter algorithm, EPIC algorithm, xCell algorithm, CIBERSORT algorithm, quanTIseq algorithm, and TIMER algorithm, further verified the stability and robustness of the ssGSEA results for two CMLBS groups.


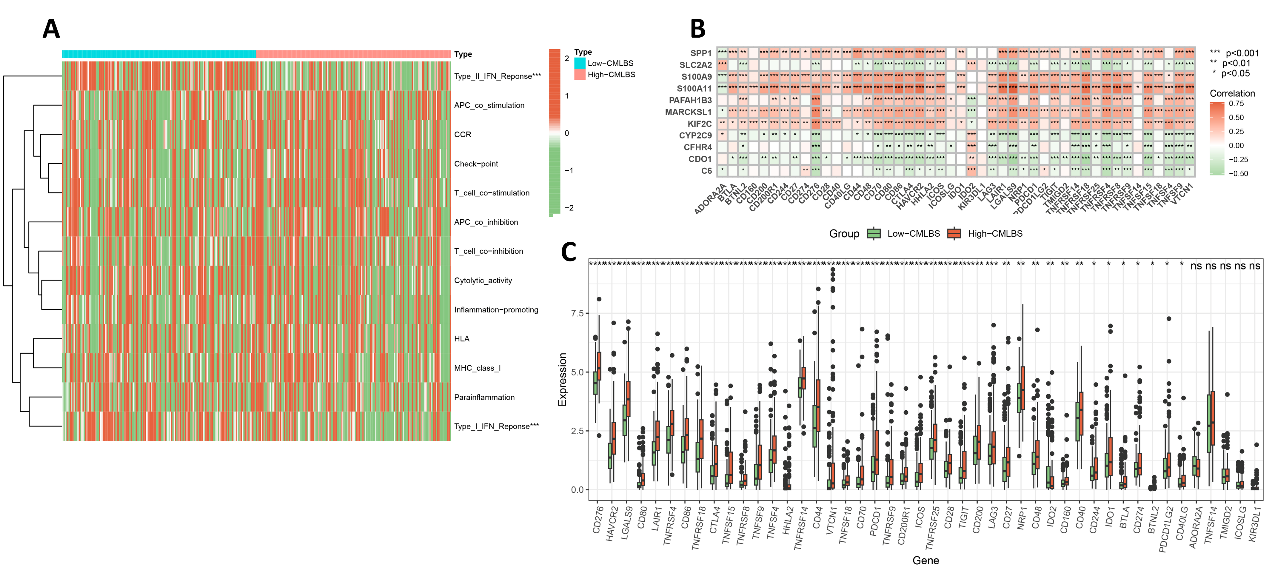


**Figure S10**. Prediction of immunotherapy efficacy. (A). Immune-related pathways’ activity showing a significant difference between the high-CMLBS group and the low-CMLBS group. (B). The 11 module genes were associated with common immune checkpoints. (C). Expression levels of immune checkpoints in high- and low-CMLBS groups.
